# Home-based, telehealth, and hybrid exercise interventions for adults undergoing or recovering from hematopoietic stem cell transplantation: a systematic review of randomized controlled trials

**DOI:** 10.3389/fresc.2026.1846935

**Published:** 2026-05-20

**Authors:** Manar Eid, Haytham Alkerdasy, Maha Metwally, Eman Mohamed Abdelwahab, Ladislav Batalik

**Affiliations:** 1 Faculty of Physical Therapy, Benha University, Benha, Egypt; 2 College of Physical Therapy, Misr University for Science and Technology, Giza, Egypt; 3 Department of Rehabilitation, University Hospital Brno, Brno, Czechia; 4 Department of Physiotherapy and Rehabilitation, Faculty of Medicine, Masaryk University, Brno, Czechia; 5 Department of Public Health, Faculty of Medicine, Masaryk University, Brno, Czechia; 6 Rehabilitation Clinic, Faculty of Medicine, Masaryk University, Brno, Czechia

**Keywords:** exercise intervention, functional capacity, hematopoietic stem cell transplantation, home-based exercise, systematic review, telehealth

## Abstract

**Background:**

Hematopoietic stem cell transplantation (HSCT) is associated with substantial treatment-related morbidity, including reduced physical function, fatigue, and impaired quality of life. Home-based and telehealth-supported exercise may improve access to rehabilitation in this population, but randomized evidence remains limited.

**Objective:**

To systematically review randomized controlled trials evaluating the effectiveness, feasibility, and safety of structured exercise interventions delivered in home-based, telehealth, or hybrid formats for adults undergoing or recovering from HSCT.

**Methods:**

A systematic search of PubMed, Scopus, Web of Science, and Cochrane CENTRAL was conducted from database inception to November 2025. Randomized controlled trials involving adults undergoing autologous or allogeneic HSCT were included if they evaluated structured exercise performed before, during, or after transplantation. Primary outcomes included physical function and physical fitness, including functional capacity, physical performance, muscle strength, gait-related measures, and fatigue. Secondary outcomes included quality of life, psychological outcomes, physical activity, feasibility, adherence, retention, safety, and adverse events.

**Results:**

Seven randomized controlled trials involving 542 adults were included. Interventions varied in timing, content, and delivery mode, and included aerobic, resistance, balance, or multimodal exercise delivered in unsupervised home-based, supervised telehealth, or hybrid formats. Across studies, the most consistent trend of improvement was observed in functional capacity and physical performance, particularly in outcomes such as the 6-minute walk test, gait-related measures, and sit-to-stand performance. Findings for muscle strength, fatigue, and quality of life were less consistent. Adherence was generally high in studies reporting it, often exceeding 80%, and serious exercise-related adverse events were uncommon.

**Conclusions:**

Structured exercise delivered in home-based, telehealth, or hybrid settings appears feasible and generally safe for adults undergoing or recovering from HSCT. Based on the limited randomized evidence available, the most consistent trend of improvement was observed in functional capacity and physical performance, whereas broader patient-reported outcomes remain less certain. Larger, methodologically robust trials are needed to clarify optimal timing, intervention design, and effects on quality of life, fatigue, and other patient-centered outcomes.

**Systematic Review Registration:**

identifier PROSPERO CRD420251245892.

## 1 Introduction

Hematopoietic stem cell transplantation (HSCT) serves as a potentially life-saving, curative, or life-prolonging intervention for numerous hematologic malignancies. Each year, approximately 70,000 HSCT procedures are conducted globally (1). The incidence of patients receiving HSCT has experienced a notable rise over recent decades (2). Despite the treatment's capacity to extend life expectancy, this is often accompanied by co-morbidities such as graft-vs.-host disease, infections, and oral mucositis, which result in years lived with disabilities as well as transplant-related mortality (3, 4). In addition, many patients undergoing HSCT procedures have adverse effects from the treatment, which include reduced physical functioning, reduced quality of life (QoL), and fatigue (5–7).

Growing evidence has emphasized the essential role of exercise in both the prevention and management of cancer (8). Targeted physical activity programs, including aerobic, resistance, and multimodal exercise interventions, have shown significant improvements in enhancing physical function, QoL, and alleviating cancer-related fatigue across various patient demographics, including those who have undergone HSCT (9, 10).

However, various hindrances often restrict patients from accessing extensive cancer rehabilitation programs. These hindrances may include socioeconomic factors, transportation problems, work commitments, financial issues, and time limitations, which often restrict patients from maintaining regular face-to-face physical activity-based interventions (11). This demonstrates the need for recent, accessible, and engaging rehabilitation programs to effectively overcome the participation hindrances (12).

Telemedicine has surfaced as a viable approach for providing specialized care from a distance (13). Through the use of electronic communication technology, telemedicine has the potential to improve access to post-HSCT care, reduce the burden of frequent in-person visits, and facilitate ongoing patient monitoring, even in the presence of unstable clinical conditions (14). While telemedicine has tremendous potential in the delivery of care for HSCT patients, its use, efficacy, and impact have not been adequately assessed. Albeit a recent scoping review mapped available evidence (11), no systematic review has synthesized the effects of home-based or telehealth exercise interventions specifically in HSCT populations. This review aims to evaluate the effectiveness, feasibility, and safety of such interventions based on randomized controlled studies.

## 2 Methods

### 2.1 Study design

This systematic review was conducted with the guidance of the PRISMA 2020 reporting guideline (15). The systematic review protocol has been registered prospectively in the International Prospective Register of Systematic Reviews (PROSPERO; registration number: CRD420251245892). The main objective of this systematic review was to synthesize evidence from randomized controlled trials (RCTs) that examined the impact of structured exercise, including aerobic, resistance, balance, and multimodal training, on functional, physical, and psychological outcomes in adult patients who were undergoing HSCT. Only studies that examined the impact of post-transplant, pre-transplant, or peri-transplant exercise interventions were included.

The conduct of the review was broadly consistent with the registered PROSPERO protocol. Minor clarifications were made during manuscript preparation. First, hybrid interventions were considered eligible when they included a substantial home-based or telehealth-supported exercise component and were not delivered exclusively as centre-based rehabilitation. Second, reference lists of included studies and relevant reviews were checked to maximize search sensitivity. Third, outcomes were narratively organized according to the domains reported across the included trials; however, all outcomes were consistent with those prespecified in PROSPERO. No changes were made to the target population, randomized study design eligibility, or overall narrative synthesis approach.

### 2.2 Search strategy

A comprehensive literature search was carried out in PubMed, Scopus, Web of Science, and Cochrane Central Register of Controlled Trials (CENTRAL) from the inception of the databases until November 2025. A combination of controlled vocabulary (MeSH terms) and keywords was used in the literature search. A comprehensive search strategy combining controlled vocabulary (MeSH terms) and free-text terms was used to identify relevant studies. The search included terms related to telehealth, rehabilitation, and hematopoietic stem cell transplantation. Broad terms (e.g., “rehab*”, “therap*”, and “home-based”) were intentionally used to maximize sensitivity and capture a wide range of exercise-based interventions, including different modalities such as walking. The full search strategies for all databases are provided in the Supplementary Material. The search strategy was adapted for the other databases. In addition, the reference lists of included studies and relevant systematic reviews were manually searched to identify additional eligible trials. Detailed search strategies for all databases are provided in the Supplementary Material. Screening and duplicate removal were performed using Rayyan systematic review software to facilitate study selection.

### 2.3 Eligibility criteria

Eligibility criteria were informed by the PICOS criteria. The target population included adult participants 18 years or older with autologous or allogeneic HSCT, regardless of the type of cancer, disease status, or treatment period. Eligible intervention included structured exercise, which could be aerobic, strength, balance, or multimodal training, conducted in supervised, unsupervised, home-based, or telehealth settings, conducted before, during, or after HSCT. The comparator could be usual care, standard treatment, delayed intervention, or education control. The primary outcomes were physical function and physical fitness, including functional capacity, physical performance, muscle strength, gait-related measures, the 6-minute walk test, peak oxygen uptake, Short Physical Performance Battery, and sit-to-stand performance, as well as fatigue assessed using validated patient-reported measures. Secondary outcomes included QoL, psychological outcomes, physical activity, feasibility, adherence, retention, safety, and adverse events. Pilot and feasibility randomized controlled trials were also included; however, these studies were interpreted with caution as they are not primarily designed to assess effectiveness.

### 2.4 Data extraction

Data extraction was performed independently by two reviewers using a standardized form to ensure consistency and minimize bias. Any disagreements were resolved through discussion. Variables extracted included the following: author, year of publication, country, sample size, demographics of the participants (age, sex, BMI, cancer type), transplant type and conditioning intensity, intervention details (type, frequency, duration, intensity, timing, and mode of delivery), details of the comparator, outcome measures, follow-up periods, adherence, retention and withdrawal, and details of the procedures for monitoring the safety of the participants.

### 2.5 Risk of bias assessment

Methodological quality of the included studies was independently assessed by two reviewers using the Cochrane Risk of Bias 2 tool (16). These domains included the following: randomization process, deviations from the intended interventions, completeness of outcome data, the validity and reliability of the outcome measures, and the selective reporting of the outcomes. These domains were assessed as being at low risk of bias, some concerns, or a high risk of bias.

Data synthesis was conducted narratively. Findings were summarized according to intervention type, timing relative to HSCT, delivery mode, and outcome domain. A meta-analysis was not performed because of the limited number of included studies and substantial clinical and methodological heterogeneity, including differences in transplant type, intervention timing, exercise content, supervision level, outcome measures, and follow-up duration.

Risk of bias due to missing results was considered as part of the assessment of selective reporting within the RoB 2 framework. Formal assessment of publication bias using funnel plots was not performed because fewer than 10 studies were included and the review was synthesized narratively. Therefore, publication bias could not be reliably assessed statistically.

## 3 Results

### 3.1 Study selection and included trials

**Figure 1:**
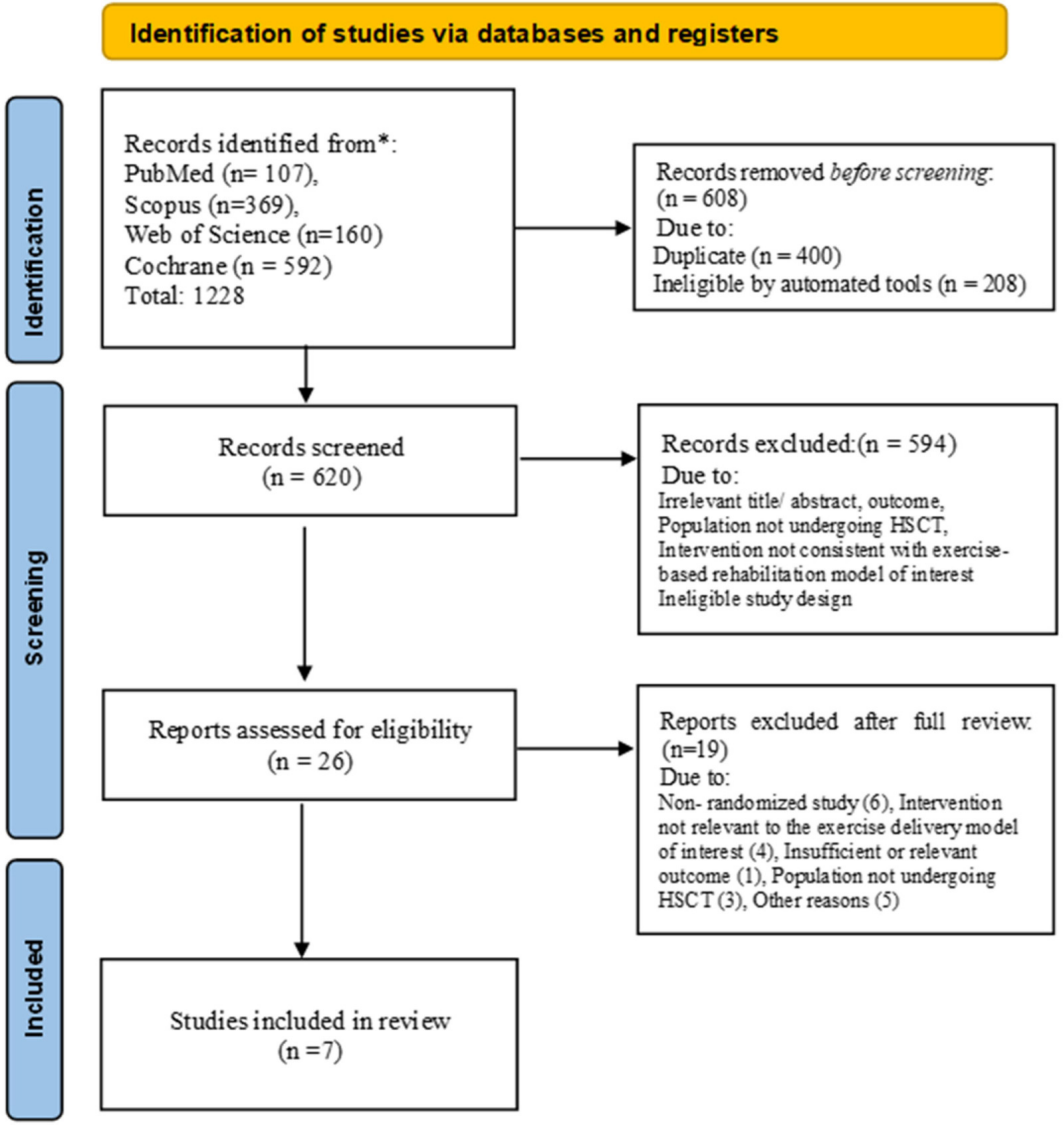
Flow chart.

The systematic search yielded 1,228 records, and 26 full text articles were screened, with 7 RCTs included (Figure 1). The trials represented a total of 542 adults with hematological malignancies (17–23). Cancer populations were heterogeneous. Sample sizes ranged between 20 and 139 participants (Mean:77). The primary focus across these trials includes evaluation of physical performance, strength, QoL, fatigue, and psychological well-being, with adherence rates typically exceeding 80%. All included studies were randomized controlled trials, including pilot and feasibility RCTs.

### 3.2 Type of exercise intervention

The interventions were predominantly multimodal, consisting of combinations of aerobic and resistance training. Specific components included endurance exercise such as walking or cycling, resistance training using simple equipment, and combined programs adapted to patients' functional status. One study also included additional components such as mindfulness-based stress management and motivational support (20). Intervention intensity ranged from moderate to high, and session duration varied from 20 to 60 min, performed 2 to 7 times per week. In six studies, exercise prescriptions were adjusted according to clinical status and safety considerations (18–23). Three studies used supervised telehealth interventions (19–21), whereas four used unsupervised home-based interventions (17, 18, 22, 23) (Table 1). Detailed FITT characteristics of the exercise interventions, including frequency, intensity, session and program duration, exercise type, and delivery/supervision mode, are summarized in Table 2.

**Table 1:** Summary findings.

Study	Population/timing	Intervention	Delivery	Comparator	Key findings
Wood et al., (17)	Adults awaiting allo-HCT; pre-transplant	Aerobic interval exercise	Home-based, weekly telehealth support	Usual care	No significant between-group differences in VO2peak or 6MWD; limited by small evaluable sample.
Wiskemann et al., (18)	Adults undergoing allo-HSCT; pre-, peri-, and post-transplant	Endurance + resistance exercise	Home-based/partly supervised hybrid model	Social contact control	Improved fatigue, physical fitness/function, and distress-related outcomes.
Lee et al., (19)	Frail/pre-frail survivors ≥2 years post-HCT; post-transplant	Balance, strength, postural control	Real-time supervised telehealth	Delayed control	Feasible with high adherence; improved gait-related outcomes, but no clear between-group differences in strength or fatigue.
Ma et al., (20)	Survivors ≥6 months post-HCT; post-transplant	Multimodal exercise + stress management + motivational support	Supervised telehealth followed by unsupervised follow-up	Usual care	Improved 6MWT and sit-to-stand performance; no significant differences in handgrip strength or QoL; no adverse events.
McCourt et al., (21)	Adults with myeloma undergoing ASCT; pre-, peri-, and post-transplant	Aerobic + resistance exercise	Hybrid/home-based model with telehealth adaptation	Usual care	Feasible intervention with potential benefits for fatigue, QoL, functional capacity, and physical activity.
Potiaumpai et al., (22)	Adults scheduled for auto/allo HSCT; pre-transplant	Aerobic + resistance prehabilitation	Unsupervised home-based	Usual care + education	High acceptability and adherence; improved 6MWT, SPPB, and chair-stand performance; no exercise-related serious adverse events.
Coleman et al., (23)	Adults with multiple myeloma receiving tandem autologous PBSCT; pre-/peri-treatment	Aerobic + resistance exercise	Home-based	Usual care	Exercise attenuated decline in walking performance during intensive treatment; more patients in usual care were unable to complete walk testing over time.

**Table 2:** FITT characteristics of the exercise interventions.

Study	Frequency	Intensity	Time	Type	Delivery/Supervision
Wood et al., (17)	3–4 sessions/week	Week 1: any intensity; from week 2: high-intensity intervals ≈80% maximal HR with lower-intensity recovery	30 min/session; 5–12 weeks	Aerobic interval exercise	Home-based with weekly telehealth support
Wiskemann et al., (18)	Pre/post-HSCT: 3 endurance + 2 resistance sessions/week; hospitalization: 5 endurance + 2 resistance sessions/week	Borg RPE 12–14 for endurance; 14–16 for resistance	20–40 min pre/post-HSCT; ≈30 min during hospitalization; program covered pre-HSCT, hospitalization, and 6–8 weeks post-HSCT	Endurance + resistance exercise	Home-based pre/post-HSCT with weekly telehealth; partly supervised during hospitalization
Lee et al., (19)	3 sessions/week	Individualized progressive resistance-band progression	30–60 min/session; 8 weeks	Dynamic balance, strength, core stability, postural control	Real-time supervised telehealth
Ma et al., (20)	Exercise 3×/week; MBSM 1×/week plus home practice during weeks 1–6	Aerobic 50%–75% HRR; resistance 65%–80% 1RM; target RPE 11–15, later adjusted by feedback	Exercise 30–60 min/session; 6-week supervised phase with follow-up to 12 months	Multimodal exercise: aerobic + resistance; plus mindfulness-based stress management and motivational support	Supervised home-based telehealth via Skype, followed by unsupervised follow-up with motivational support
McCourt et al., (21)	3 sessions/week	Individualized aerobic intensity 60%–80% HR	Pre-HSCT 6–8 weeks; during hospitalization; post-HSCT 12 weeks. Sessions progressed from 15 min to ≥30 min pre-HSCT; ≤30 min during/post-HSCT	Aerobic + resistance exercise	Hybrid/home-based model; face-to-face or Zoom-based telehealth adaptation; weekly telephone support during/post-HSCT
Potiaumpai et al., (22)	Resistance 5×/week; aerobic 5–7×/week	Resistance RPE 14–16/20; aerobic RPE 12–14/20	Resistance 30–45 min/session; aerobic ≈ 15 min/session; 2–24 weeks until hospital admission	Aerobic + resistance prehabilitation	Unsupervised home-based exercise
Coleman et al., (23)	Resistance training on alternate days	Moderate intensity; Borg scale used during exercise assessments	Continued from baseline through intensive treatment phase, ≈15 weeks	Aerobic + resistance exercise using resistance bands	Home-based exercise

FITT, frequency, intensity, time, and type; HSCT, hematopoietic stem cell transplantation; HR, heart rate; HRR, heart rate reserve; MBSM, mindfulness-based stress management; RPE, rating of perceived exertion; 1RM, one-repetition maximum.

#### 3.2.1 Timing relative to HSCT

The timing of interventions varied substantially across studies. Pre-transplant prehabilitation initiated 2 to 8 weeks before HSCT admission was evaluated in five studies (17, 18, 21–23). Peri-transplant or early post-transplant interventions, initiated during hospitalization or soon after discharge, were reported in four studies (18–21). Two studies also included longer-term follow-up assessments at 6 or 12 months to evaluate maintenance of effects (18, 20).

### 3.3 Telehealth and home-based delivery mode

Four studies (18–21) used telehealth-supported interventions, most commonly in supervised formats delivered via videoconferencing or remote monitoring platforms. Several studies also incorporated unsupervised home-based components supported by written materials, telecoaching, or symptom-guided progression. Across studies, these delivery models were generally feasible, with high adherence and low rates of serious adverse events.

### 3.4 Risk of bias assessment

**Figure 2:**
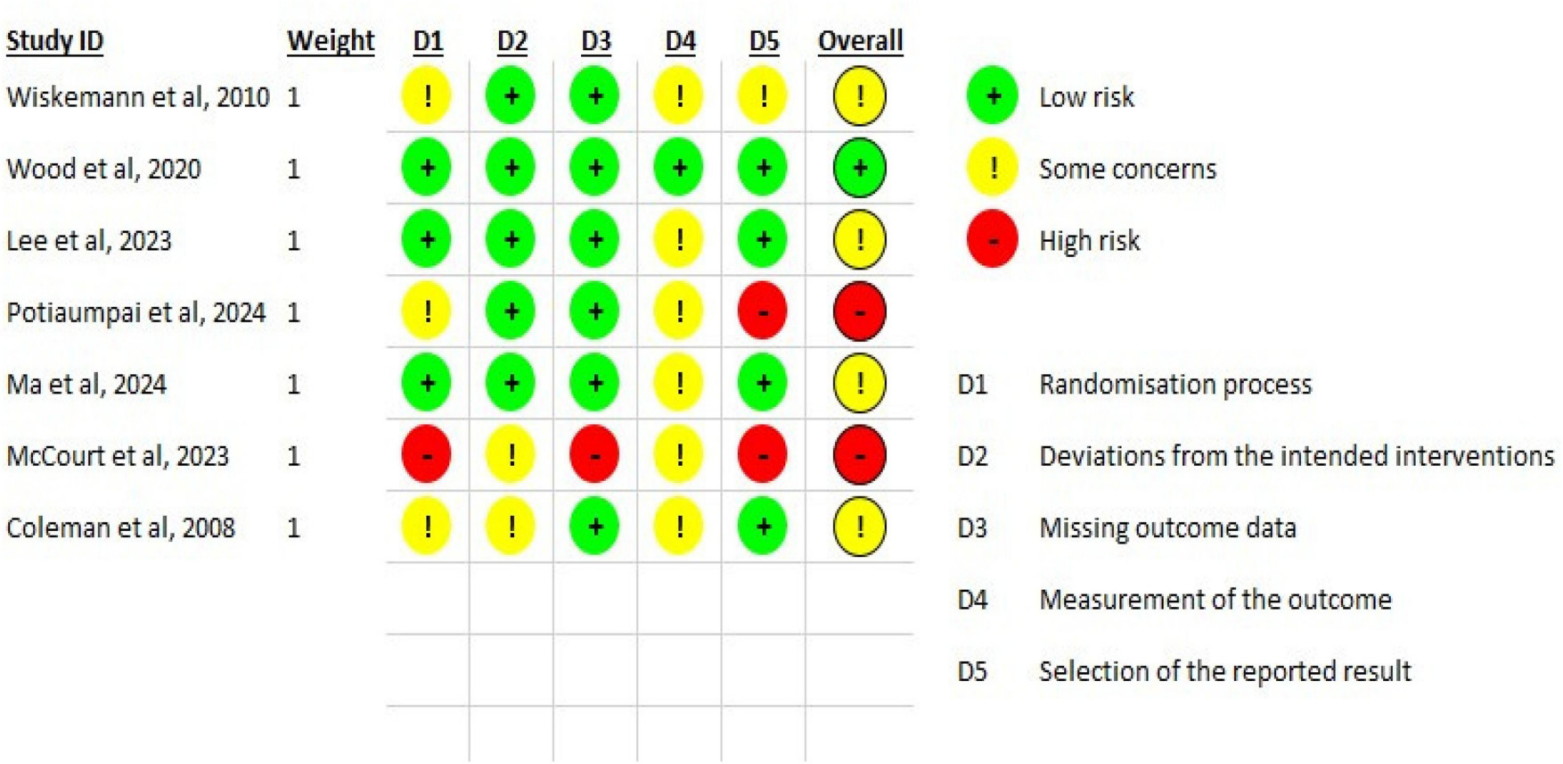
Risk of bias assessment of included studies.

The risk-of-bias assessment showed a heterogeneous methodological profile across the included trials. Wood et al. (17) was judged as low risk of bias. Ma et al. (20), Wiskemann et al. (18), Coleman et al. (23), and Lee et al. (19) were judged as having some concerns, mainly related to issues in the randomization process (Potiaumpai et al., McCourt et al.), deviations from intended interventions (Lee et al.), and reporting issues (Wiskemann et al.) (Figure 2). Potiaumpai et al. (22) and McCourt et al. (21) were judged as high risk of bias, particularly because of concerns related to randomization, missing outcome data, and reporting. Overall, the methodological limitations of several studies should be considered when interpreting the findings.

### 3.5 Effect of exercise interventions on key outcomes

#### 3.5.1 Muscle strength

Muscle strength was assessed in six studies (17–22), most commonly using handgrip dynamometry and sit-to-stand-based measures. Overall, the evidence suggests that exercise may help preserve or improve some aspects of muscle performance, although the findings were not consistent across all studies. For example, Ma et al. reported no significant between-group differences in handgrip strength despite improvements in functional capacity. Studies such as Wiskemann et al. (18) and McCourt et al. (21) suggest that hospitalization, treatment burden, and transplant-related symptoms may partly explain why improvements in strength outcomes were less consistent than improvements in functional capacity (18, 21).

#### 3.5.2 QoL

QoL was assessed in several studies using instruments such as FACT-BMT, EORTC QLQ-C30, SF-36, and distress-related measures (18, 20, 21, 23). Overall, exercise interventions appeared to attenuate decline or support recovery in quality-of-life-related outcomes, although findings were not uniform across studies. Ma et al. (20) did not identify significant between-group differences in QoL, whereas McCourt et al. (21) and Coleman et al. (23) suggested favorable trends in selected patient-reported outcomes.

#### 3.5.3 Physical activity level

Three studies assessed physical activity using step counts or self-report measures such as the International Physical Activity Questionnaire (18, 20, 21). Overall, exercise interventions appeared to support maintenance or improvement in physical activity levels, although the evidence remained limited and inconsistent. Two studies (18, 20) reported favorable changes, whereas hospitalization and treatment-related symptoms may have contributed to reductions in activity in others.

#### 3.5.4 Physical capacity

Physical capacity was examined in six studies, most commonly using the 6-minute walk test and peak oxygen uptake (17, 18, 20–23). Across studies, exercise interventions generally showed the most consistent positive signal in this outcome domain. Ma et al. reported significant improvements in 6minute walk distance, whereas Wood et al. found no statistically significant between-group differences in VO2peak or 6MWD, likely reflecting the limited evaluable sample (17). Coleman et al. (23) suggested that exercise may attenuate decline in walking performance during intensive treatment, rather than produce large absolute gains (23). Taken together, the findings suggest a possible favorable effect of exercise on physical capacity, although the magnitude of benefit varies by intervention and study context.

#### 3.5.5 Psychological well-being and fatigue

Two studies assessed psychological well-being using measures such as DASS or POMS (18, 20), and four studies evaluated fatigue. Overall, the findings suggest that exercise may help reduce fatigue and improve aspects of psychological well-being, although the results were not fully consistent across studies. Wiskemann et al. reported favorable effects on fatigue and global distress, while McCourt et al. suggested that better adherence to exercise was associated with improved fatigue and QoL (18, 21). However, the small sample sizes and heterogeneity of measures limit firm conclusions in this domain.

#### 3.5.6 Adherence and safety

Adherence was generally high in the studies that reported it (18–21, 23), often exceeding 80%, and exercise-related adverse events were rare. Safety monitoring typically included symptom review, perceived exertion, progression rules, and medical clearance procedures. These findings support the feasibility and tolerability of structured exercise in HSCT populations when appropriate monitoring and individualized adaptation are applied.

## 4 Discussion

This systematic review synthesized randomized evidence on structured exercise interventions delivered in home-based, telehealth, or hybrid formats for adults undergoing or recovering from HSCT. However, the heterogeneity across studies, small sample sizes, and the presence of studies with some concerns or high risk of bias limit the strength of these findings and preclude firm conclusions regarding effectiveness. Overall, the available evidence suggests that these interventions are feasible and generally safe, with the most consistent trend of improvement observed in functional capacity and physical performance. This pattern is broadly concordant with the wider HSCT exercise literature, in which exercise more reliably improves functional recovery than broader patient-reported outcomes, especially when trials are small and interventions are heterogeneous (11, 24–26). However, direct comparability across studies was limited by substantial clinical and methodological heterogeneity, including differences in transplant type, intervention timing, exercise characteristics, supervision level, outcome measures, and follow-up duration.

One plausible explanation is that functional outcomes such as the 6-minute walk test, gait speed, chair stand performance, or the Short Physical Performance Battery may be more responsive to short-term exercise exposure than global quality-of-life or fatigue scales. In HSCT populations, QoL and fatigue are influenced by multiple concurrent factors, including conditioning toxicity, infection risk, graft vs.-host disease, nutritional compromise, sleep disturbance, and prolonged physical deconditioning. As a result, functional outcomes may respond earlier than broader patient-reported outcomes. This interpretation is consistent with previous meta-analytic data showing favorable exercise effects on lower-limb strength, functional capacity, fatigue, and QoL, but less consistent effects on cardiorespiratory fitness, upper-limb strength, and psychosocial outcomes (24, 25). In addition, the presence of studies with some concerns and high risk of bias further limits the strength of the conclusions and suggests that the findings should be interpreted with caution. It is important to note that several included studies were pilot or feasibility trials, which are typically not powered to detect effectiveness outcomes. Therefore, their findings should be interpreted primarily in terms of feasibility and acceptability rather than definitive efficacy.

The timing of intervention is also likely to be a major source of heterogeneity. The studies included in our review covered pre-transplant prehabilitation, peri-transplant or hybrid models, and post-transplant survivorship interventions. These phases should not be interpreted as clinically equivalent. Pretransplant exercise may optimize physiological reserve before an expected decline, whereas posttransplant exercise more often aims to reverse deconditioning, frailty, and reduced activity levels. Peri-transplant programs may help attenuate decline across the full transplant trajectory. Earlier meta-analysis suggested that exercise initiated before transplantation may yield more favorable effects on upper- and lower-limb strength, fatigue, and QoL than exercise started only after transplant (24). This view is also supported by individual studies included in our review, such as prehabilitation-focused designs and peri-transplant hybrid interventions (17, 21, 22).

A key contribution of the present review is its focus on home-based and telehealth-supported delivery. This is particularly relevant in HSCT care, where patients frequently face barriers to repeated participation in center-based rehabilitation, including immunosuppression, travel burden, fatigue, scheduling demands, and the need for close symptom monitoring. This broader relevance is also supported by a recent randomized trial in lymphoma survivors, in which telehealth-supported home-based rehabilitation showed no statistically significant 12-month differences vs. matched centre-based rehabilitation for cardiorespiratory fitness and most secondary outcomes; however, these findings were not interpreted as proof of equivalence or non-inferiority (27). Recent telemedicine-focused evidence suggests that remotely supported exercise interventions are generally feasible, acceptable, and safe, although the field remains dominated by small prospective and feasibility-oriented studies (11). Across these studies, telemedicine-facilitated exercise was associated with improvements in VO2peak, 6 min walk distance, handgrip strength, and sit-to-stand performance, while also helping overcome access barriers (11, 15). Our findings are therefore clinically meaningful because they support the continued development of remote and hybrid rehabilitation models in HSCT rather than reliance on exclusively face-to-face formats (11, 15, 31).

Safety requires equally careful interpretation. In our review, adherence was frequently high and serious exercise-related adverse events were uncommon, but exercise in HSCT should not be framed as a simple yes-or-no issue. Rather, safe prescription requires individualized adaptation according to transplant phase, blood counts, infection status, symptom burden, and overall clinical stability. Recent evidence in hematologic cancer rehabilitation suggests a shift away from absolute contraindications based solely on laboratory values toward adaptation of exercise and activities of daily living according to the full clinical picture (28–30). In the scoping review of safe blood count thresholds, platelet-related decisions increasingly favored modification rather than blanket cessation, hemoglobin thresholds more often guided adaptation or temporary restriction, and neutropenia primarily required infection-control precautions rather than automatic exercise prohibition (28, 29). This supports a practical model of HSCT rehabilitation based on symptom-guided progression, monitoring of exertion and vital signs, and flexible supervision intensity (28–31).

The trajectory of recovery after HSCT is another important consideration. Recovery is often prolonged and incomplete, and short intervention periods may underestimate the potential value of exercise. In a recent longitudinal study of allo-HSCT recipients, handgrip strength and 6-minute walk performance recovered to pre-transplant levels by one year, whereas muscle mass did not fully recover and several quality-of-life domains remained below population norms (32). These data help explain why some trials show preservation of function, attenuation of decline, or faster recovery rather than large short-term gains across all domains. In HSCT populations, exercise may therefore be important not only for producing improvement, but also for limiting deterioration and accelerating return toward baseline (32, 33).

Older studies from the broader HSCT rehabilitation literature also provide useful context for interpreting our findings. Pilot and early post-transplant studies suggested that individualized or home-based aerobic exercise could improve fatigue, aerobic fitness, and physical well-being, even though many of these designs lacked a control group (34, 35). Similarly, randomized inpatient or peri-transplant studies demonstrated that structured walking or multimodal supervised exercise could improve functional status, muscle strength, aerobic capacity, and selected aspects of physical or emotional well-being, with stronger signals often seen in less fit patients or those with lower baseline function (36, 37). Together, these studies reinforce the idea that the strongest and earliest benefits of exercise in HSCT are often seen in functional recovery rather than in broader psychosocial endpoints.

The clinical implications of our review are therefore practical. First, structured exercise in HSCT does not need to rely exclusively on center-based delivery. Simple home-based prescriptions, supervised telehealth sessions, and hybrid models appear workable across different phases of care. Second, patient selection and tailoring are likely critical. Frail survivors, deconditioned patients, and those with poorer baseline functional capacity may derive substantial benefit, but may also require closer monitoring and more individualized progression (15, 36). Third, multimodal rehabilitation approaches that integrate exercise with education, behavioral support, and where appropriate nutritional support may be especially relevant in this population, given the interaction between functional decline, symptom burden, and prolonged recovery (33, 38).

Future research should move beyond small proof-of-concept trials toward adequately powered multicenter randomized studies that distinguish more clearly between pre-transplant, peri-transplant, and post-transplant rehabilitation models. Greater consistency is needed in intervention reporting, progression rules, safety monitoring, adherence metrics, and clinically relevant core outcomes. Separate analyses for autologous and allogeneic populations, as well as frail or high-risk subgroups, would also improve interpretability (39). Based on the limited randomized evidence available, the present review suggests that structured exercise may represent a promising supportive care strategy in HSCT, particularly for preserving or restoring functional capacity; however, stronger evidence is needed before firm conclusions can be drawn regarding broader patient-centered outcomes (11, 24–26, 32).

### 4.1 Limitations

This review has several limitations. Only seven randomized trials were included, and several were pilot or feasibility studies with small sample sizes. In addition, the restriction to English-language publications and the exclusion of grey literature may have introduced selection bias. The studies differed substantially in transplant type, disease characteristics, intervention timing, supervision level, and outcomes assessed. In addition, some interventions were multimodal, which limits attribution of effects to exercise alone. Finally, although the review focused on home-based and telehealth-supported exercise, some included studies also involved hybrid or partly supervised delivery models, which should be acknowledged when interpreting the findings.

## 5 Conclusion

Structured exercise interventions delivered in home-based, telehealth, or hybrid settings appear feasible and generally safe for adults undergoing or recovering from HSCT. Based on the limited randomized evidence available, the most consistent trend of improvement was observed in functional capacity and physical performance, whereas effects on QoL, fatigue, and muscle strength were less consistent. These findings should be interpreted cautiously because of the small number of trials, heterogeneity of interventions and outcomes, and methodological concerns in several studies. Larger, methodologically robust trials with clearer reporting of timing, safety, adherence, and patient-centered outcomes are needed.
