# Supplementary material for: Home-based, telehealth, and hybrid exercise interventions for adults undergoing or recovering from hematopoietic stem cell transplantation: a systematic review of randomized controlled trials
Source: Front Rehabil Sci. 2026 May 20;7:1846935. doi: 10.3389/fresc.2026.1846935 (PMC13230005; doi:10.3389/fresc.2026.1846935)
Supplement: Supplementary file 1 [file Datasheet1.docx]

Search Strategy

Pubmed 107

("Telemedicine"[Mesh] OR telemed*[tiab] OR tele-med*[tiab] OR telehealth*[tiab] OR tele-health*[tiab] OR telerehab*[tiab] OR tele-rehab*[tiab] OR teleconsult*[tiab] OR tele-consult*[tiab] OR tele-coach*[tiab] OR telecoach*[tiab] OR tele-care*[tiab] OR telecare*[tiab] OR tele-screen*[tiab] OR telescreen*[tiab] OR tele-therap*[tiab] OR teletherap*[tiab] OR tele-diagnos*[tiab] OR telediagnos*[tiab] OR tele-mentor*[tiab] OR telementor*[tiab] OR telepsyc*[tiab] OR tele-psyc*[tiab]) OR (("Videoconferencing"[Mesh] OR teleco*[tiab] OR tele-co*[tiab] OR telemetr*[tiab] tele-metr*[tiab] OR tele-home*[tiab] OR telehome*[tiab] OR videoconf*[tiab] OR video-conf*[tiab] OR web-based[tiab] OR internet-based[tiab] home-based[tiab] OR Google Meet[tiab] OR Cisco Webex[tiab] OR Microsoft Teams[tiab] OR remote*[tiab]) AND ("therapy" [Subheading] OR "Therapeutics"[Mesh] OR rehab*[tiab] OR therap*[tiab] OR treatment*[tiab] OR consult*[tiab] OR care OR monitor*[tiab] OR counsel*[tiab])) AND ("Hematopoietic Stem Cell Transplantation"[Mesh] OR (hematopoietic[tiab] OR haematopoietic[tiab]) AND transplant*[tiab]))

Scopus 369

( TITLE-ABS-KEY ( telemedicine OR telemed* OR tele-med* OR telehealth* OR tele-health* OR telerehab* OR tele-rehab* OR teleconsult* OR tele-consult* OR tele-coach* OR telecoach* OR tele-care* OR telecare* OR tele-screen* OR telescreen* OR tele-therap* OR teletherap* OR tele-diagnos* OR telediagnos* OR tele-mentor* OR telementor* OR telepsyc* OR tele-psyc* ) ) OR ( TITLE-ABS-KEY ( videoconferencing OR teleco* OR tele-co* OR telemetr* OR tele-metr* OR tele-home* OR telehome* OR videoconf* OR video-conf* OR "web-based" OR "internet-based" OR "home-based" OR "google meet" OR "cisco webex" OR "microsoft teams" OR remote* ) AND TITLE-ABS-KEY ( rehab* OR therap* OR treatment* OR consult* OR care OR counsel* ) ) TITLE-ABS-KEY ( "Hematopoietic Stem Cell Transplantation" ) OR ( TITLE-ABS-KEY ( hematopoietic OR haematopoietic ) AND TITLE-ABS-KEY ( transplant* ) )

Cochrane central 592

("telemedicine" OR "telemed" OR "telehealth" OR "telerehab" OR "teleconsult" OR "telecoach" OR "telecare" OR "telescreen" OR "teletherapy" OR "telediagnosis" OR "telementor" OR "telepsych" OR "videoconferencing" OR "telemonitor" OR "home-based" OR "web-based" OR "internet-based" OR "remote") AND (therapy OR therapeutics OR rehabilitation OR treatment OR consult OR care OR monitor OR counsel) AND ("Hematopoietic Stem Cell Transplantation" OR hematopoietic OR haematopoietic OR transplant)

Web of Science 160

TS=(telemedicine OR telemed* OR tele-med* OR telehealth* OR tele-health* OR telerehab* OR tele-rehab* OR teleconsult* OR tele-consult* OR tele-coach* OR telecoach* OR tele-care* OR telecare* OR tele-screen* OR telescreen* OR tele-therap* OR teletherap* OR tele-diagnos* OR telediagnos* OR tele-mentor* OR telementor* OR telepsyc* OR tele-psyc*) OR TS=(videoconferencing OR teleco* OR tele-co* OR telemetr* OR tele-metr* OR tele-home* OR telehome* OR videoconf* OR video-conf* OR "web-based" OR "internet-based" OR "home-based" OR "Google Meet" OR "Cisco Webex" OR "Microsoft Teams" OR remote*) AND TS=(therapy OR therapeutics OR rehab* OR therap* OR treatment* OR consult* OR care OR monitor* OR counsel*)

TS= ("Hematopoietic Stem Cell Transplantation") OR (TS= (hematopoietic OR haematopoietic) AND TS=(transplant*))
